# Complications of nephrogenic systemic fibrosis following repeated exposure to gadolinium in a man with hypothyroidism: a case report

**DOI:** 10.1186/1752-1947-5-566

**Published:** 2011-12-07

**Authors:** Arpita Aggarwal, Allison A Froehlich, Paulina Essah, Nooshin Brinster, Whitney A High, Robert W Downs

**Affiliations:** 1 Department of Internal Medicine, Virginia Commonwealth University, PO Box 980111, Richmond, Virginia 23298, USA; 2 Division of Endocrinology and Metabolism, Virginia Commonwealth University, PO Box 980111, Richmond, Virginia 23298, USA; 3 Department of Dermatopathology, Virginia Commonwealth University, PO Box 980111, Richmond, Virginia 23298, USA; 4 Department of Dermatology & Pathology, University of Colorado Health Sciences Center, 1999 North Fitzsimons Parkway, Bioscience East, Suite 120, Aurora, CO 80045, USA

## Abstract

**Introduction:**

Nephrogenic systemic fibrosis is a condition that has recently been recognized in patients with chronic renal disease and is associated with use of gadolinium-based contrast agents of ubiquitous use in magnetic resonance imaging scans. The condition is believed to arise through inadequate renal clearance of the gadolinium-based contrast agents, resulting in bodily deposition of the gadolinium; this is most widely recognized in the skin, but also occurs in other tissues.

**Case presentation:**

We report the case of a 52-year-old Caucasian man with hypothyroidism and chronic renal disease who developed nephrogenic systemic fibrosis upon repeated exposure to gadolinium, and who presented with a subsequent malabsorption of levothyroxine. This malabsorption resolved only partially upon amelioration of other conditions that might contribute to malabsorption, including edema and infectious diarrhea. The presence of gadolinium was quantified in specimens from his gastrointestinal tract. Our patient otherwise demonstrated adequate gastrointestinal nutritive absorption, objectively shown by normal albumin levels, resolution of diarrhea, and maintenance of his bodily weight.

**Conclusions:**

Our observations suggest that nephrogenic systemic fibrosis can also affect tissue of the gastrointestinal tract, potentially contributing to partial malabsorption of levothyroxine in patients with hypothyroidism.

## Introduction

Nephrogenic sclerosing fibrosis (NSF), also known as nephrogenic sclerosing dermopathy, is characterized as thickening and hardening of the skin overlying extremities and trunk secondary to fibrosis of the dermis in patients with renal failure [1,2]. The condition was first described by Cowper *et al. *as an idiopathic cutaneous fibrosing disorder or scleromyxedema-like illness in patients on dialysis in whom transplantation had been unsuccessful [3]. Other than end-stage renal disease (ESRD) patients on dialysis, patients with hepatorenal syndrome (HRS), recent vascular surgery, recent venous thromboembolic events, and those with an estimated glomerular filtration rate (GFR) < 30 ml/minute/1.73 m^2 ^appear at greatest risk for developing NSF [4,5]. NSF was initially believed to be limited to the skin, but recent reports, including autopsies, have identified involvement of skeletal muscle, myocardium, lungs, and kidneys, indicating the systemic nature of this disease [2,6,7].

## Case presentation

A 52-year-old Caucasian man with chronic kidney disease (CKD) was admitted to our university hospital from a skilled nursing facility with generalized edema, woody thickening of his skin, and bilateral hand contractures after recurrent hospitalizations during which time he underwent multiple MRI scans with gadolinium-based contrast agents (GBCA). Our patient's medical history was significant for type 2 diabetes mellitus, hypertension, CKD secondary to diabetic nephropathy, hypothyroidism, congestive heart failure, and hemochromatosis. Upon admission, his medications included neutral protamine Hagedorn (NPH) insulin 15 units twice daily, levothyroxine (LT4) 75 μg once daily, clonidine 0.1 mg twice daily, metoprolol sustained-release 25 mg once daily, amlodipine 5 mg once daily, enteric-coated aspirin 325 mg daily, and calcitriol 0.5 μg once daily.

In the four months prior to this admission, he was first admitted for aspiration pneumonia and had a prolonged hospital course. He was started with ceftriaxone and metronidazole, later changed to piperacillin/tazobactam and levofloxacin for broader coverage. His baseline creatinine level was 3.5 mg/dL and he had a glomerular filtration rate (eGFR) of 24 mL/minute. Shortly after admission, he developed acute kidney failure (ARF). Acute tubular necrosis (ATN) and increased creatinine was attributed to episodes of hypotension. Over the course of several days, his creatinine level stabilized to a range between 4.0 and 4.5 mg/dL. His hospital course was then complicated by bloody diarrhea, prompting several gastrointestinal studies. A computed tomography (CT) scan of his abdomen revealed a diffusely fluid-filled colon with colitis. Flexible sigmoidoscopy showed acute and chronic inflammation with biopsy results consistent with ischemic colitis. Thereafter, he underwent magnetic resonance angiography (MRA) and MRI with gadolinium of the abdomen to evaluate for mesenteric vein thrombosis. These tests returned negative results. Our patient was subsequently treated with total parenteral nutrition (TPN) for bowel rest and his symptoms resolved.

Subsequently, our patient developed fungemia. An abdominal MRI scan with gadolinium revealed no abscess. At this time, his creatinine level increased to 6.6 mg/dL and his glomerular filtration rate (eGFR) decreased to 9 mL/minute. Our patient was treated for presumptive recurrent ischemic colitis without perforation and was continued on a prolonged course of TPN for bowel rest. His symptoms improved and he was discharged to a skilled nursing facility.

**Figure 1:**
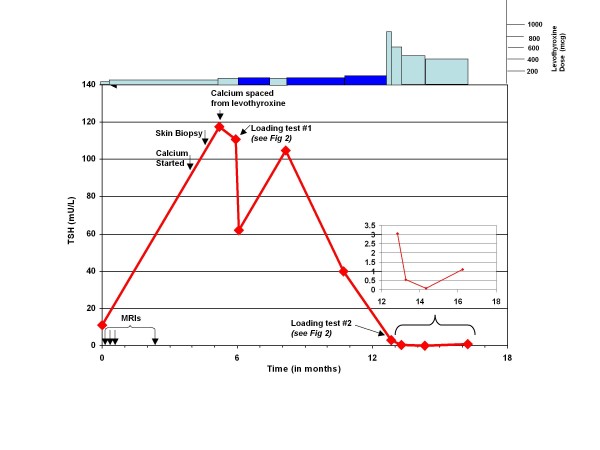
**Change in serum TSH as a function of the time from our patient's initial hospitalization**. Light blue boxes above graph, oral levothyroxine with coincident doses noted by markings on upper right. Dark blue boxes above graph, intra-muscular levothyroxine with coincident doses noted by markings on upper right. Black arrows in left bottom corner, dates of MRI scans with the gadolinium contrast. Inset graph illustrates magnified view of TSH trend after restarting oral levothyroxine treatment. IM, intra-muscular; TSH, thyroid stimulating hormone; Tx, treatment.

Shortly after his discharge, he was readmitted with new right-sided weakness and underwent an MRI scan with GBCA that revealed a left thalamic cerebrovascular accident, from which he recovered quickly. A new physical finding revealed skin with a diffuse woody peau d'orange appearance with tightening of the palmar fascia bilaterally at both hands with all fingers slightly drawn in. His neurological examination results were unremarkable. His admission thyroid stimulating hormone (TSH) level was 11.21 mU/L with a free thyroxine level (T4) of 0.7 ng/dL while on 75 μg of levothyroxine (LT4) (Figure 1).

A skin biopsy was performed for evaluation of the hand contractures. The histology revealed numerous dermal fibroblast-like cells with dermal mucin deposition, consistent with NSF. Two weeks later, thyroid studies revealed a serum TSH rise to 117.5 mU/L and free T4 drop to 0.4 ng/dL. His LT4 dose was increased from 75 to 100 μg daily. Our patient was observed by nursing staff for his medication compliance.

**Figure 2:**
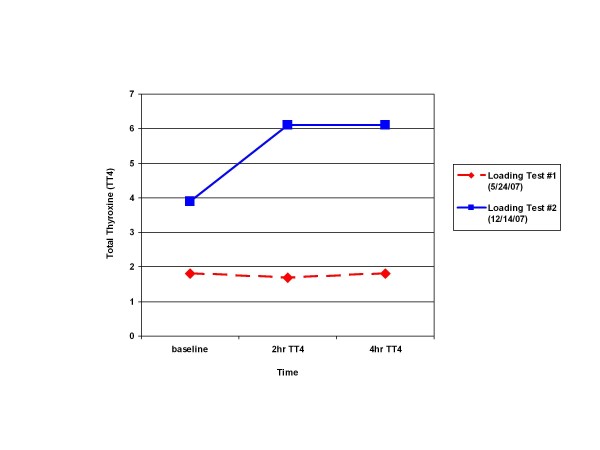
**Change in total thyroxine as a function of time during the oral levothyroxine loading tests**. Red diamonds with dashed line, loading test 1; blue squares with solid line, loading test 2.

Because of the possibility of LT4 malabsorption, our patient underwent an oral LT4 loading test (Figure 2). The protocol for this test entailed obtaining baseline total T4, TSH, and free T4 levels, followed by administration of a single oral dose of LT4 1000 μg, with repeat total T4 measured at two hours, and again at four hours after LT4 administration [8]. Baseline laboratory tests showed a total TSH level of 110.6 mU/L, free T4 level of < 0.3 ng/dL and total T4 level of 1.8 ng/dL. The challenge test revealed a failure of total T4 to rise, with values of 1.7 ng/dL at two hours and 1.8 ng/dL at four hours, consistent with LT4 malabsorption. His LT4 malabsorption was suspected to be partly secondary to NSF caused by exposure to gadolinium from the multiple MRI scans.

Our patient was started on intravenous LT4 with some improvement of symptoms. The results of repeat laboratory testing four days later revealed a decline in TSH of 61.95 mU/L and rise in total T4 to 4.4 ng/dL. He was discharged on intra-muscular LT4 100 μg daily. However, two months later, our patient was seen at an outside community hospital for treatment of a urinary tract infection (UTI) and was switched back to oral LT4 upon discharge. Testing one month later showed a recurrent rise in TSH to 104.83 mU/L, and our patient was restarted on intra-muscular LT4 at a dose of 100 μg daily. His pharmacy was contacted to ensure compliance with the prescribed refill. Repeat laboratory tests results from two months later showed modest improvements, with a TSH of 39.82 mU/L. Based on these results, his intra-muscular LT4 was increased to 120 μg daily.

Of note, during clinical follow-up, our patient did not appear to have malabsorption of other nutrients. His albumin rose over time from 2.5 g/dL on initial admission up to 3.7 g/dL 10 months later. Also, his diarrhea did not recur, and he maintained his weight.

Four years ago, because of a nationwide shortage of intra-muscular LT4, a decision was made to repeat the oral loading LT4 test to help assess whether bowel edema, as a complication of hypothyroidism, had been the primary etiological factor in the malabsorption of LT4. The results of this test showed a total T4 level of 3.9 ng/dL, which rose to a value of 6.1 ng/dL for both the two-hour and four-hour time intervals. With this small, but demonstrable, amount of oral LT4 absorption, a decision was made to do a retrial of oral LT4, starting at a dose of 900 μg daily. Based on repeat laboratory analysis, his LT4 dose was gradually decreased to a maintenance dose of 400 μg daily. He maintained his TSH on oral LT4 for the next year until his death secondary to sudden cardiac arrest.

**Figure 3:**
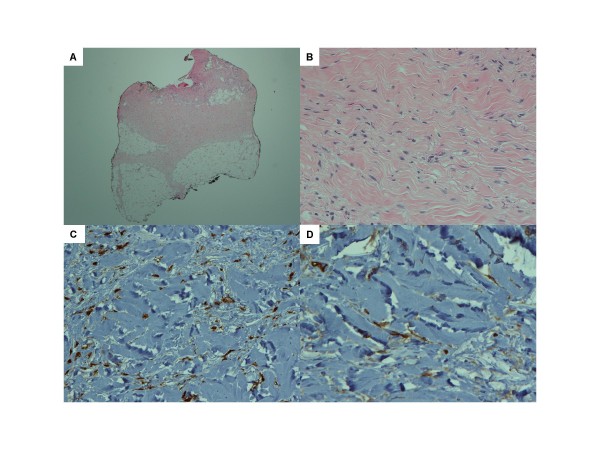
**(A) Lower dermis and subcutaneous fat with widened and thickened fibrous septae and increased dermal fibroblastic proliferation with thickened collagen; (B) increased dermal fibroblastic proliferation with thickened collagen fibers; (C, D) immunohistochemistry stains of dermal fibroblastic cells are positive for factor 13a (C) and CD34 (D)**.

Because we suspected gastrointestinal involvement of nephrogenic fibrosing dermopathy as a contributing factor in this partial malabsorption, gadolinium quantification was performed on gastrointestinal specimens at the laboratory of one of the authors (WAH). This analysis revealed 154.0 parts per million (PPM) in the skin, 22.8 PPM in the colon, and 7.0 PPM in the ileum/cecum/colonic specimens with all appropriate positive and negative control tissue blanks functioning (Figure 3).

## Discussion

To the best of our knowledge, this case presentation is the first to describe a gastrointestinal involvement of NSF that appeared to result in uncontrolled hypothyroidism secondary to malabsorption of oral LT4. Of particular interest, our patient did not appear to have malabsorption of other oral medications. LT4 is known to be predominantly absorbed in the small intestine, specifically the jejunum and ileum. Ischemic colitis, infectious diarrhea and use of TPN during his hospitalization, along with the presence of diffuse bowel edema, may have each contributed to poor absorption of this medication. However, in such circumstances, one would expect the malabsorption to resolve with improvement in each of these conditions; in our patient's case, malabsorption persisted despite resolution of these other complicating conditions.

The issue of potential bowel edema contributing to malabsorption was addressed by utilizing an intra-muscular formulation of LT4, to allow for attainment of a euthyroid state. However, repeat oral loading LT4 testing demonstrated that our patient remained considerably resistant to absorption, despite achieving normalized TSH levels and resolution of visible bodily edema. These findings led us to the theoretical consideration that NSF might have intestinal mucosa involvement. This theory is supported by the presence of demonstrable gadolinium in gastrointestinal biopsies, along with his continued need for supratherapeutic doses of LT4 to maintain a euthyroid state. However, it is critical to note that the presence of gadolinium in the tissue does not necessarily imply pathogenicity, particularly as the gastrointestinal specimen did not show any other significant pathology. Admittedly, there is limited data on gadolinium levels in bodily tissues of patients with NSF. Disturbances in LT4 absorption have been reported with co-administration of calcium carbonate, ferrous sulfate, aluminum hydroxide, chromium picolinate and magnesium, prompting speculation that other divalent and trivalent metal ions could interfere with absorption [9-12]. It is unclear but certainly worthy of speculation that gadolinium, being a trivalent ion, could interfere with thyroxine absorption, but it is further speculative as to whether this would be a persistent anomaly, considering that gadolinium so deposited in tissue most likely exists as precipitated gadolinium phosphate [13]. These are intriguing avenues for future exploration.

NSF has been described in various organs, including the myocardium, lungs, kidneys, testes and dura mater [2,6,7]. There are several hypotheses regarding the pathogenesis of NSF. Some investigators assert that transforming growth factor β is involved in the pathogenesis of NSF [6]. *In vitro *studies have shown that transforming growth factor β1 has the potential to induce fibrocyte differentiation and to promote expression of fibrocyte collagen I, which deposits in the cells to cause fibrosis [14].

The US Food and Drug Administration (FDA) first notified health care professionals and the public about the gadolinium-related risks for NSF five years ago. GBCAs are commonly used to improve visualization of structures when patients undergo an MRI or MRA procedure. Five gadolinium-based contrast agents were approved for use in the US at the time of this report, and include: Magnevist (gadopentetate dimeglumine), Ominiscan (gadodiamide), OptiMARK (gadoversetamide), MultiHance (gadobenate dimeglumine), and Prohance (gadoteridol). When a specific agent was identified, Omniscan (the gadolinium agent used in our patient) was the most often implicated in NSF, followed by Magnevist and OptiMARK. Approximately three years ago, a sixth agent, Vasovist (gadofosveset trisodium), was approved by the FDA for use in MRA.

There is no consistently effective treatment for NSF, although improvement has been reported with plasmapheresis, and in most reported cases, the disease appears to correspond to the improvement in underlying renal function [15].

## Conclusions

As general internists and endocrinologists continue to care for more patients with chronic kidney disease and hypothyroidism, it is prudent that physicians recognize any emerging disorders in these patients, including any untoward complications associated with NSF. Continued observation and additional reporting will be necessary to ascertain if NSF, with deposition of gadolinium in the bowel, results in malabsorption of LT4 or other medications, and what mechanism of malabsorption may be involved in this phenomenon.

## Consent

Written informed consent was obtained from the patient's next-of-kin for publication of this case report and any accompanying images. A copy of the written consent is available for review by the Editor-in-Chief of this journal.

## Competing interests

The authors declare that they have no competing interests.

## Authors' contributions

AA was the primary clinical physician for our patient. AF acted as an endocrinology resident, providing insight into the symptoms displayed by our patient. PE and RD were endocrinologists who also provided analysis and interpretation of the data from our patient. NB and WH were dermatologists who assessed the pertinent symptoms of our patient. WH was also responsible for the pathology slides displayed in this report. All authors contributed to and approved the final version of the manuscript.
